# Effects of total urethral suspension combined with posterior pelvic floor reconstruction on sexual function in women with stress urinary incontinence and concomitant vaginal laxity syndrome

**DOI:** 10.3389/fsurg.2025.1643253

**Published:** 2025-08-07

**Authors:** Yuan Li, Jihong Shen, Ling Li, Daoming Tian, Yubin Wen, Hongcheng Li, Jiangna Gu, Qian Luo, Zhenhua Gao, Xingqi Wang

**Affiliations:** Department of Urology, The First Affiliated Hospital, Kunming Medical University, Kunming, China

**Keywords:** pelvic floor dysfunction (PFD), pelvic organ prolapse (POP), stress urinary incontinence—SUI, vaginal laxity syndrome, female sexual dysfunction (FSD), pelvic organ prolapse/urinary incontinence sexual function questionnaire short form

## Abstract

**Objective:**

To assess the impact of total urethral suspension with posterior pelvic floor reconstruction on sexual function in women with stress urinary incontinence(SUI) and concomitant vaginal laxity syndrome (VLS), including partner satisfaction.

**Methods:**

Clinical data from 150 pelvic floor dysfunction patients were collected at the First Affiliated Hospital of Kunming Medical University (March 2023–March 2024). Preoperative assessments included demographics, obstetric/surgical history, menopausal status, sexual activity and **maximum levator hiatal area during Valsalva maneuver on ultrasound**. Seventy-five sexually active patients completed the Pelvic Organ Prolapse/Urinary Incontinent Sexual Function Questionnaire Short Form (PISQ-12), with partner satisfaction and vaginal tightness evaluations. At the 1-year postoperative follow-up, patients underwent outpatient clinical evaluations, including the PISQ-12 questionnaire, assessments of vaginal tightness and partner satisfaction, and pelvic floor ultrasound measurements.

**Results:**

The subjective cure rate for urinary incontinence was 86.67% with a 10.67% improvement rate. Significant improvements were observed in PISQ-12 scores (preoperative: 17.56 ± 5.56 vs. postoperative: 11.72 ± 4.23; *P* < 0.01). Partners reported increased overall satisfaction (3.21 ± 0.92–3.81 ± 1.06; *P* < 0.01) and enhanced perception of vaginal tightness (2.47 ± 0.58–4.17 ± 0.62; *P* < 0.01).

**Conclusion:**

The combined surgical procedure demonstrates significant therapeutic efficacy in managing SUI and concomitant VLS, with postoperative outcomes showing substantial improvements in both urinary continence and sexual function. Total urethral suspension provides comprehensive to treat SUI. Posterior pelvic floor reconstruction restores anatomical integrity by reducing the levator hiatus and reconstructing the perineal body, thereby normalizing vaginal axis alignment. The subsequent vaginal tightening achieved through these procedures significantly enhances sexual function for both patients and their partners.

## 1 Introduction

Current research remains inadequate regarding the impact of pelvic floor dysfunction (PFD) and its surgical treatments on female sexual function, resulting in limited evidence-based guidance for clinicians developing individualized treatment plans (1). Existing surgical approaches remain confined to single-disease treatment paradigms, failing to incorporate the pathological mechanisms of both SUI and VLS into comprehensive therapeutic strategies. This limitation directly compromises complete symptom resolution and overall quality of life improvement.

To address this clinical gap, the present study evaluates patients with concurrent SUI and VLS undergoing combined total urethral suspension and posterior pelvic floor reconstruction. Pre- and postoperative comparisons are conducted using the PISQ-12, male partner satisfaction scores, and vaginal tightness assessments to determine the clinical efficacy of this combined surgical approach in improving sexual function for both patients and their partners.

## 2 Material and methods

Clinical data were collected from patients diagnosed with SUI and concomitant VLS who underwent total urethral suspension combined with posterior pelvic floor reconstruction at the Department of Urology, First Affiliated Hospital of Kunming Medical University between March 2023 and March 2024. Inclusion criteria comprised: ①clinical diagnosis of SUI with concurrent stage I or II anterior pelvic organ prolapse; ② regular sexual activity (≥3 instances/month); ③ confirmed VLS diagnosis; and ④voluntary participation with signed informed consent. Exclusion criteria included: ① presence of urge incontinence or mixed urinary incontinence; ② refusal to undergo sexual function assessments or inability to complete long-term follow-up; ③ history of pelvic floor surgeries or urogenital disorders; ④ diagnosed psychiatric conditions; or ⑤ concurrent cervical elongation syndrome.This investigation strictly adhered to the Declaration of Helsinki and received ethical approval from the Institutional Review Board of First Affiliated Hospital of Kunming Medical University (Approval No. L33-2022). Written informed consent was obtained from all participants after comprehensive explanation of study objectives, methodologies, and potential risks.

### 2.1 Surgical indications and methods

**Figure 1:**
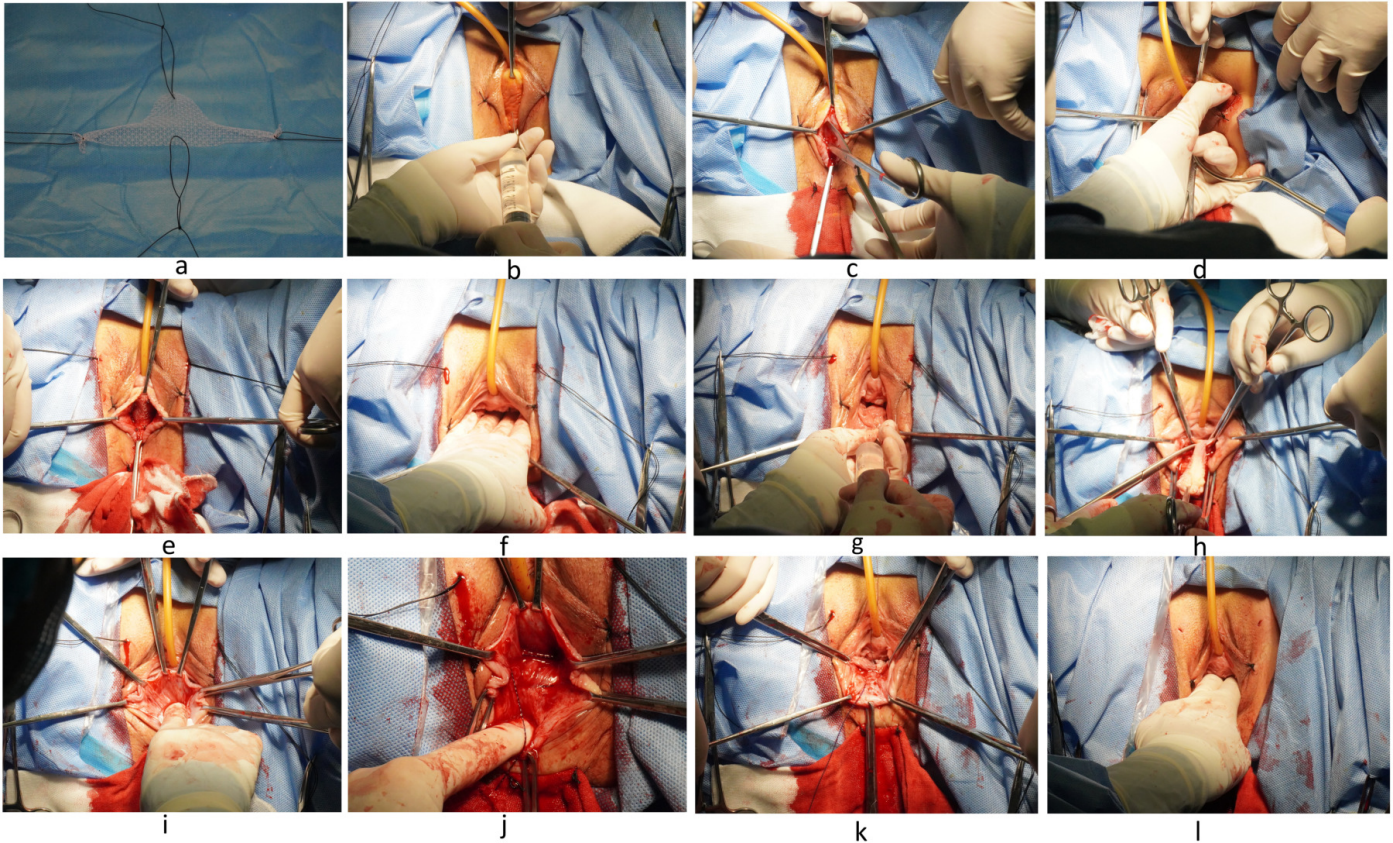
**(a)** single-arm mesh trimming. **(b)** The anterior vaginal wall hydrodissection. **(c)** Separation of the urethrovaginal space. **(d)** Puncture introducer-guided mesh placement. **(e)** Mesh fixation. **(f)** Genital hiatus measurement. **(g)** Rectovaginal hydrodissection. **(h)** The posterior vaginal wall diamond-shaped flap. **(i)** Dissection the rectovaginal space. **(j)** Suturing of bilateral levator ani muscles and pararectovaginal fascia. **(k)** Suturing of the perineal body and external anal sphincter. **(l)** Genital hiatus measurement.

The single-arm mesh is specifically indicated for patients with SUI complicated by stage I–II anterior pelvic prolapse. Intraoperatively, the four-arm polypropylene mesh is individually tailored based on pelvic floor anatomical requirements (Figure 1a), with meticulous positioning to ensure full contact along the total urethra to 1–2 cm anterior to the bladder neck. All surgical procedures were performed by a single experienced surgeon, Dr. Shen, to maintain technical consistency throughout the study.

(1) After the anesthesia takes effect, the patient is placed in the lithotomy position. Routine disinfection and sterile draping are performed, followed by insertion of a 16F indwelling urethral catheter.
(2) Following adequate hydrodissection of the anterior vaginal wall (Figure 1b), a midline incision is initiated 1 cm distal to the external urethral orifice and extended caudally to the bladder neck. Dissection is performed between the urethra and bilateral vaginal walls (Figure 1c), advancing posteriorly to the descending pubic rami.
(3) A 2-mm incision is created 0.5 cm lateral to the superior border of the left inferior pubic ramus. The surgeon's right index finger is positioned posterior to the descending pubic ramus, guiding the insertion of a tunneler at a 45-degree oblique angle through the incision (Figure 1d). This allows the pelvic floor mesh suspension arm to rotate along the descending pubic ramus and emerge posterior to the pubic bone. The identical procedure is replicated on the contralateral side. Cystoscopic examination is then performed to confirm proper mesh placement spanning from the total urethra to the bladder neck. Four anchoring points of the mesh are secured using 2-0 absorbable surgical sutures (Figure 1e). Continuous 3-0 barbed suture for submucosal tissue approximation with bladder neck mucosal plication, and 2-0 absorbable sutures for anterior vaginal wall repair.
(4) The genital hiatus is measured and recorded (Figure 1f). Following hydrodissection in the rectovaginal space superior to the pelvic diaphragm, the subdiaphragmatic space, and bilateral vaginal sulci (Figure 1g), a diamond-shaped posterior vaginal wall flap is designed with its narrowest segment corresponding to the pelvic diaphragm (Figure 1h). The flap is elevated cephalad to the cervical plane. The rectovaginal space is dissected until the levator ani muscle bundles become visible (Figure 1i). Continuous #0 absorbable sutures are placed to approximate the bilateral levator ani muscles and paravaginal fascia (Figure 1j), effectively reducing the levator hiatus area. Sequential vaginal narrowing is achieved from the cervical plane to the pelvic diaphragm level, establishing the posterior vaginal angle. The anterior vaginal segment is closed using 2-0 absorbable sutures.
(5) Continuous #0 absorbable sutures are placed to approximate the subdiaphragmatic perineal body and external anal sphincter complex (Figure 1k), completing the perineal reconstruction. The posterior vaginal wall is subsequently reconstituted using 2-0 absorbable sutures, with final vaginal calibration confirming adequate patency accommodating two surgical fingerbreadths (Figure 1l). The use of the image has been authorized by the patient.

### 2.2 Diagnostic criteria for vaginal laxity syndrome

①Subjective sensation of vaginal laxity (2).②Physical examination:The patient is positioned in lithotomy. With adequate lubrication, the examiner's fingers are inserted into the vaginal canal at rest until no discomfort is reported. Vaginal laxity is graded based on finger accommodation: Normal: Two fingers fitting snugly; Mild: Two fingers with loose fit; Moderate: Three fingers; Severe: Four or more fingers (3). A diagnosis of vaginal laxity is confirmed when >2 fingers are accommodated during this examination.

### 2.3 Observation metrics

Eligible patients completed the PISQ-12 preoperatively and at 1-year follow-up. The PISQ-12 consists of 12 items scored on a 1–5 Likert scale, with higher scores indicating worse sexual function (4). This validated instrument evaluates sexual frequency, satisfaction, and dysfunction domains. Male partners were administered a two-item questionnaire assessing overall sexual satisfaction and perceived vaginal tightness, with each item rated from 1 (lowest) to 5 (highest). Higher scores reflect greater satisfaction. All follow-up questionnaires were administered either face-to-face during clinical visits, consistently conducted by the same trained researcher. To ensure data quality, the researcher provided standardized, non-leading explanations using plain language to clarify questionnaire items without influencing responses. Participants were given sufficient time to comprehend each item and complete the questionnaires independently. Responses were recorded anonymously.

### 2.4 Statistical methods

The data were analyzed using SPSS Statistics software (version 27.0). Normally distributed continuous variables are presented as mean ± standard deviation, with between-group comparisons performed using paired-sample t-tests. For non-normally distributed continuous variables, data are expressed as median values, and between-group comparisons were conducted using the nonparametric Wilcoxon rank-sum test. A two-tailed *P* < 0.05 was considered statistically significant.

## 3 Result

### 3.1 General information and therapeutic efficacy

This study evaluated 75 patients undergoing total urethral suspension combined with posterior pelvic floor reconstruction. The procedure demonstrates significant efficacy and favorable safety outcomes (Table 1). At 1-year follow-up, the subjective cure rate is 86.67% (65 patients with complete continence), with 10.67% reporting occasional mild stress incontinence, yielding an overall effectiveness rate of 97.34%. The treatment failure rate is 2.67%.

**Table 1:** General information and therapeutic efficacy.

Parameter	Value
Age(years), mean ± SD[range]	43.88 ± 7.2
BMI(kg/m^2^), mean ± SD[range]	23.60 ± 2.64
Parity, median	2 (1,2)
Vaginal deliveries, median	2 (1,2)
Postoperative follow-up period (moths), mean ± SD[rang]	13.08 ± 1.15
SUI Outcomes	*n* (%)
Objective cure rate	65 (86.67)
Subjective improvement	8 (10.67)
Treatment failure	2 (2.67)

Objective cure: negative standardized stress test at 1-year follow-up.

Subjective improvement: occasional stress incontinence during vigorous activity.

Treatment failure: symptom improvement or worsening.

### 3.2 Complications and management

Mesh exposure occurs in 5.3% of cases, with all cases successfully managed by partial mesh removal and topical estrogen therapy, showing no recurrence. This rate is consistent with the reported 4%–10% polypropylene mesh erosion rate in literature (5, 6). Vaginal irritation is observed in 2.67% of patients, both associated with suture exposure and resolved completely after suture removal. Dyspareunia is reported in 1.33% of cases, with symptoms significantly improved following baclofen treatment.

### 3.3 Sexual function outcomes

PISQ-12 evaluation reveals (Table 2): ① No significant change is observed in female sexual desire postoperatively (*P* = 0.083), though substantial improvement is noted in urinary incontinence- and prolapse-related sexual interference (decreased scores in items 6–9, *P* < 0.001). ② The total score decreases from 17.56 ± 5.67 to 11.72 ± 4.23 (*P* < 0.001), indicating enhanced sexual confidence and overall sexual function improvement. Male partners report significantly increased satisfaction, with tightness scores improving from 2.47 ± 0.58 to 4.17 ± 0.62 (*P* < 0.001). The levator hiatal area significantly decreased from 23.91 ± 7.79cm² to 20.38 ± 5.56cm²(*P* < 0.001). Dyspareunia is reported in 2.67% of cases, which is successfully managed through regular intercourse with lubricant use.

**Table 2:** Preoperative and postoperative sexual function outcomes and ultrasound data.

Parameter	Preoperative	postoperative	t	*p*
PISQ-12 items				
Q1:sexual desire frequency	2.09 ± 0.77	2.05 ± 0.77	−1.76	0.083
Q6:coital incontinence	1.17 ± 0.98	0.15 ± 0.43	−9.88	<0.001
Q7:fear of incontinence during intercousre	1.39 ± 1.27	0.31 ± 0.49	−8.91	<0.001
Q8:avoidance due to prolapse	1.09 ± 1.18	0.28 ± 0.56	−7.17	<0.001
Q9:negative emotion during intercourse	1.11 ± 0.89	0.33 ± 0.55	−8.77	<0.001
Total PISQ-12 score	17.56 ± 5.67	11.72 ± 4.23	−13.30	<0.001
Partner-reported outcomes				
Overall satisfaction	3.21 ± 0.92	3.81 ± 1.06	3.89	<0.001
Perceived tightness	2.47 ± 0.58	4.17 ± 0.62	19.73	<0.001
Levator hiatus area (cm^2^)	23.91 ± 7.79	20.38 ± 5.56	−5.75	<0.001

Sexual desire frequency: “How often do you feel sexual desire? This may include interest in sexual activity, planning for sex, or frustration due to lack of sex”.

Coital incontinence: “Do you experience urine leakage during intercourse?”.

Fear of incontinence: “Does concern about fecal/urinary incontinence inhibit your sexual activity?”.

Prolapse avoidance: “Do you avoid intercourse due to vaginal bulge symptoms?”.

Negative emotions: “Do you experience fear, disgust, or guilt during partnered sex?”.

^a^ PISQ-12 Item Definitions.

### 3.4 Special case report

Vaginal lubrication disorders are observed in 2.67% of female patients, potentially associated with altered tissue elasticity or estrogen fluctuations (7). The etiology remains undetermined due to patient refusal of additional diagnostic workup. Persistent vaginal foreign body sensation is reported by 1.33% of male partners. After exclusion of mesh exposure, this condition is considered neuroadaptive dysfunction, warranting long-term monitoring if persistent.

## 4 Discussion

The total urethral suspension combined with posterior pelvic floor reconstruction enables comprehensive three-dimensional management of SUI through dual mechanisms of anatomical restoration and biomechanical optimization. The key rationale is as follows: ① The central segment of the single-arm mesh is tailored to the urethral length, ensuring full urethral coverage and support while effectively correcting urethral hypermobility. Compared to conventional slings, the single-arm mesh demonstrates a broader and more uniform pressure distribution, which prevents urethral obstruction and subsequent voiding dysfunction—a finding supported by the absence of postoperative voiding difficulty in follow-up evaluations. Additionally, the wider arms of the single-arm mesh provide increased contact area with the descending pubic ramus, resulting in enhanced fixation stability and reduced recurrence rates attributable to mesh migration. ② Posterior pelvic floor reconstruction focuses on repairing the ruptured levator ani muscle. Restoration of muscular continuity significantly reduces the transverse diameter of the levator hiatus, while anatomical repositioning of the levator plate reestablishes dynamic mid-vaginal support to the bladder base, effectively addressing urinary incontinence. ③ Correction of the vaginal axis to its physiological inclination (the 130° angle between the mid and lower vagin a (8) provides structural reinforcement to the urethra, minimizes bladder neck mobility, and optimizes abdominal pressure distribution, thereby restoring biomechanical equilibrium in pelvic-abdominal pressure transmission. This integrated approach achieves a triple mechanism of anti-incontinence efficacy in SUI management.

Analysis of sexual desire frequency (PISQ-12 Question 1) reveals no statistically significant difference between preoperative (2.09 ± 0.77) and postoperative (2.05 ± 0.77) scores (*P* = 0.083), suggesting limited clinical impact of the procedure on female libido. This finding contrasts with results reported by Saida et al. (9, 10), a discrepancy that may be attributed to methodological differences. Importantly, Saida's study incorporated heterogeneous surgical interventions (vaginal hysterectomy, pelvic floor reconstruction, and mid-urethral sling procedures), potentially compromising result comparability through three distinct mechanisms: First, pelvic floor anatomical modifications vary by surgical approach; second, technique-dependent differential effects on neurovascular bundles are observed; third, disparities in recovery timelines and complication profiles may confound sexual function evaluations.

The Keziban team (11) demonstrated through a randomized controlled clinical trial that the combination of TOT (transobturator tape) surgery with perineoplasty yields superior clinical outcomes in sexual function improvement compared to TOT surgery alone.

This study analyzes core physiological and psychological indicators of sexual function in PFD patients using the PISQ-12 questionnaire, comparing dynamic changes in key domains pre- vs. postoperatively. The surgical approach is demonstrated to indirectly facilitate psychological rehabilitation through symptom alleviation following physiological improvement. Significant improvements are observed across all measured parameters:Physiological domain (Question 6) scores decrease from 1.17 ± 0.98 to 0.15 ± 0.43 (*P* < 0.01), indicating consistent therapeutic efficacy in reducing coital incontinence and establishing a biological foundation for psychological improvement (12). Incontinence-related avoidance behaviors (Question 7) show reduction from 1.39 ± 1.27 to 0.31 ± 0.49 (*P* < 0.01), suggesting marked alleviation of anticipatory anxiety regarding sexual activity (13, 14). Prolapse-related avoidance (Question 8) decreases from 1.09 ± 1.18 to 0.28 ± 0.56 (*P* < 0.01), demonstrating that anatomical reconstruction effectively reduces body image-related anxiety (15). Negative sexual emotions (Question 9) improve from 1.11 ± 0.89 to 0.33 ± 0.55 (*P* < 0.01), confirming that symptom resolution significantly mitigates secondary psychological distress. The observed reductions in psychological domain scores indicate stable population-level psychotherapeutic effects. This physiological-psychological cascade is mediated through: ① anatomical reconstruction enabling symptom relief, ② subsequent body image enhancement fostering confidence, and ③ consequent establishment of a virtuous cycle of sexual participation. These findings provide a novel multidimensional framework for evaluating pelvic floor reconstruction outcomes, emphasizing the need for clinicians to consider both direct symptomatic relief and indirect psychological benefits in postoperative assessment.

The study results demonstrate significant postoperative improvement in partners' sexual function parameters. Male overall satisfaction scores increase from 3.21 ± 0.92 to 3.81 ± 1.06 (*P* < 0.01), while vaginal tightness ratings improve from 2.47 ± 0.58 to 4.17 ± 0.62 (*P* < 0.01), indicating that the procedure positively impacts both patient outcomes and partners’ sexual experience.

This therapeutic effect is achieved through three anatomical mechanisms: ① the perineal body and levator ani muscles are reconstructed, ② vaginal axis restoration is accomplished with mid-vaginal support, and ③ vaginal length is increased while reducing the lower vaginal transverse diameter. These modifications collectively enhance penile-vaginal contact surface area and frictional coefficients during intercourse, thereby improving male sexual pleasure.

This study has several limitations that warrant consideration. The modest sample size (*n* = 75) may limit statistical power and generalizability, though ongoing enrollment aims to address this. The relatively short follow-up period restricts long-term outcome assessment; extended follow-up with 24-month interval analyses is underway. As a single-center study, our findings require multi-center validation. The lack of a control group precludes direct comparisons, underscoring the need for controlled studies to confirm these observations.

## 5 Conclusion

This combined surgical approach appears to offer several potential clinical benefits: (1) it may effectively improve SUI, (2) it could facilitate anatomical and functional restoration of pelvic floor structures in patients with concomitant VLS, and (3) it might contribute to enhanced sexual quality of life for both patients and their partners. The procedure seems to address key treatment goals for pelvic floor disorders (PFD), while potentially providing a comprehensive surgical option for patients with concurrent female sexual dysfunction (FSD).

## Data Availability

The original contributions presented in the study are included in the article/Supplementary Material, further inquiries can be directed to the corresponding author.

## References

[1] Verbeek M Hayward L. Pelvic floor dysfunction and its effect on quality of sexual life. Sex Med Rev. (2019) 7(4):559–64. 10.1016/j.sxmr.2019.05.007 31351916

[2] Polland A Fitzgerald JJ Iwamoto A Furuya RL Duong V Bradley SE DEVELOPS: description of vaginal laxity and prolapse and correlation with sexual function. Am J Obstet Gynecol. (2020) 222(3):S779–80. 10.1016/j.ajog.2019.12.050 PMC8766263 34629323

[3] Gaye T Merdan S Toygar U Dincer A. Patient reported vaginal laxity, sexual function and stress incontinence improvement following vaginal rejuvenation with fractional carbon dioxide laser. J Plast Surg Hand Surg. (2021) 55(1):25–31. 10.1080/2000656X.2020.1828897 33030095

[4] Zhu L Yu S Xu T Yang X Lu Y Lang J. Validation of the Chinese version of the pelvic organ prolapse/urinary incontinence sexual questionnaire short form (PISQ-12). Int J Gynecol Obstet. (2012) 116(2):117–9. 10.1016/j.ijgo.2011.08.021 22079198

[5] Baessler K Maher CF. Mesh augmentation during pelvic-floor reconstructive surgery: risks and benefits. Curr Opin Obstet Gynecol. (2006) 18(5):560–6. 10.1097/01.gco.0000242961.48114.b0 16932053

[6] Xiuli S Xiaowei Z Jianliu W. Surgical outcomes and quality of life post-synthetic mesh-augmented repair for pelvic organ prolapse in the Chinese population. J Obstet Gynaecol Res. (2014) 40(2):509–14. 10.1111/jog.12167 24118430

[7] Wang C-L Long C-Y Juan Y-S Liu C-M Hsu C-S. Impact of total vaginal mesh surgery for pelvic organ prolapse on female sexual function. Int J Gynecol Obstet. (2011) 115(2):167–70. 10.1016/j.ijgo.2011.05.019 21839998

[8] Yinluan O Fan L Rui W Wanwan X Weizeng Z Weijia Y A simplified method for evaluating the anatomical axis of the upper two-thirds of the vagina on MRI: a hospital-based cross-sectional study. Gynecol Obstet Clin Med. (2023) 3(4):229–35. 10.1016/j.gocm.2023.10.003

[9] Saida A Raheela M Huda S. Surgery for pelvic organ prolapse and stress urinary incontinence and female sexual functions: a quasi-experimental study. Pak J Med Sci. (2021) 37(4):1099–103. 10.12669/pjms.37.4.3892 34290790 PMC8281190

[10] Pardo J Solà V Ricci P. Colpoperineoplasty in women with sensation of a wide vagina. J Minim Invasive Gynecol. (2009) 16(6S):S47–S8. 10.1016/j.jmig.2009.08.175

[11] Doğan K Öztoprak MY Dura MC Aslan İÖ. The effect of stress incontinence and pelvic organ prolapse surgery on sexual function and quality of life. J Turk Ger Gynecol Assoc. (2024) 25(2):96–101. 10.4274/jtgga.galenos.2024.2023-1-13 38869033 PMC11576632

[12] Stadnicka G Łepecka-Klusek C Pilewska-Kozak A Jakiel G. Psychosocial problems of women with stress urinary incontinence. Ann Agric Environ Med. (2015) 22(3):499–503. 10.5604/12321966.1167723 26403124

[13] Clark SMG Huang Q Sima AP Siff LN. Effect of surgery for stress incontinence on female sexual function. Obstet Gynecol Surv. (2020) 75(6):346–7. 10.1097/01.ogx.0000668324.04671.67 31923066

[14] Zhang Y Song X Kang J Ma Y Ma C Zhu L. Sexual function after tension-free vaginal tape procedure in stress urinary incontinence patients. Menopause. (2020) 27(10):1143–7. 10.1097/GME.0000000000001583 32986394

[15] Shicong L Tongxiang D Wei Z Samuel S Zhipeng Z Maolin H Sexual functions in women with stress urinary incontinence after mid-urethral sling surgery: a systematic review and meta-analysis of prospective randomized and non-randomized studies. J Sex Med. (2020) 17(10):1956–70. 10.1016/j.jsxm.2020.07.003 32741744

